# Lipoprotein biosynthesis as a target for anti-*Wolbachia *treatment of filarial nematodes

**DOI:** 10.1186/1756-3305-3-99

**Published:** 2010-10-14

**Authors:** Kelly L Johnston, Bo Wu, Ana Guimarães, Louise Ford, Barton E Slatko, Mark J Taylor

**Affiliations:** 1 Filariasis Research Laboratory, Molecular and Biochemical Parasitology Group, Liverpool School of Tropical Medicine, Liverpool, L3 5QA, UK; 2 Molecular Parasitology Division, New England Biolabs Inc., Ipswich, Massachusetts 01938 USA

## Abstract

**Background:**

Lymphatic filariasis and onchocerciasis are debilitating diseases caused by filarial nematodes. Disease pathogenesis is induced by inflammatory responses following the death of the parasite. *Wolbachia *endosymbionts of filariae are potent inducers of innate and adaptive inflammation and bacterial lipoproteins have been identified as the ligands that bind toll-like receptors (TLR) 2 and TLR6. Lipoproteins are important structural and functional components of bacteria and therefore enzymes involved in *Wolbachia *lipoprotein biosynthesis are potential chemotherapeutic targets.

**Results:**

Globomycin, a signal peptidase II (LspA) inhibitor, has activity against Gram-negative bacteria and a putative *lspA *gene has been identified from the *Wolbachia *genome of *Brugia malayi *(*w*Bm). The amino acids required for function are strictly conserved and functionality was verified by complementation tests in a temperature-sensitive *Escherichia coli lspA *mutant. Also, transformation of wild type *E. coli *with *Wolbachia lspA *conferred significant globomycin resistance. A cell-based screen has been developed utilizing a *Wolbachia*-containing *Aedes albopictus *cell line to assay novel compounds active against *Wolbachia*. Globomycin was screened using this assay, which resulted in a dose-dependent reduction in *Wolbachia *load. Furthermore, globomycin was also effective in reducing the motility and viability of adult *B. malayi in vitro*.

**Conclusions:**

These studies validate lipoprotein biosynthesis as a target in an organism for which no genetic tools are available. Further studies to evaluate drugs targeting this pathway are underway as part of the A-WOL drug discovery and development program.

## Background

Lymphatic filariasis and onchocerciasis are leading causes of global morbidity, with 150 million people afflicted and 1.5 billion people at risk. The filarial nematodes responsible for these diseases; *Wuchereria bancrofti*, *Brugia malayi *(lymphatic filariasis) and *Onchocerca volvulus *(onchocerciasis), have a mutualistic association with a bacterial endosymbiont, *Wolbachia pipientis*. The symbiotic relationship is essential for parasite growth, development, fecundity and survival [1].

*Wolbachia *have emerged as a novel target for antibiotic therapy to treat filariasis. Antibiotic studies using *ex vivo *and *in vivo *animal model systems (reviewed by [1]) and extensive field trials have demonstrated the effectiveness of antibiotics such as doxycycline in the treatment of filariasis [2-14]. Depletion of *Wolbachia *leads to long-term sterility and ultimately to the death of adult worms. Furthermore, *Wolbachia*-targeted treatment has also been shown to lead to a reduction in the severity and improvement of lymphoedema and hydrocoele pathology in lymphatic filariasis [3,5]. Despite these clear benefits of doxycycline therapy over conventional treatments, the extended period of treatment and contraindication in children under eight and pregnancy compromises their delivery through mass drug treatment programmes. This has driven the formation of the A-WOL (anti-*Wolbachia*) consortium to discover and develop new drugs active against *Wolbachia *for the treatment of filariasis that would be compatible with current control programme strategies.

*Wolbachia *lipoproteins have emerged as potent stimulators of the inflammatory pathogenesis of filarial disease [15]. Genomic analysis indicates that *Wolbachia *contain the lipoprotein biosynthesis genes *lgt *and *lspA *but not *lnt*, N-acyltransferase, which is required for the triacylation of apolipoproteins. This suggests that *Wolbachia *lipoproteins cannot be triacylated and accounts for the recognition by the diacyl-lipoprotein receptor complex TLR2/6 [15]. Bioinformatic and database searches consistently predicted the presence of only three lipoproteins in *Wolbachia*: Peptidoglycan-associated lipoprotein (PAL), a Type IV Secretion System protein (VirB6) and Small protein A [15].

Lipoproteins are important structural and functional components of bacteria and their biosynthesis is essential for bacterial viability. Globomycin, an inhibitor of lipoprotein signal peptidase, LspA [16], has previously been demonstrated to have potent anti-bacterial activity against Gram-negative bacteria [17-20]. We therefore sought to test whether globomycin was active against *Wolbachia *and to validate lipoprotein biosynthesis as a *Wolbachia *drug target.

## Results

### Identification and verification of a functional *w*Bm *lspA *gene

**Figure 1:**
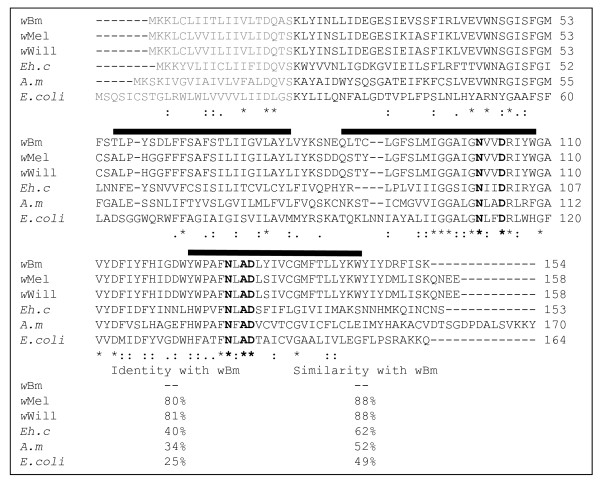
**Sequence alignment of LspA proteins**. Sequence alignment of LspA proteins from *Wolbachia *of *Brugia malayi *(*w*Bm, AAW71005), *Wolbachia *of *Drosophila melanogaster *(*w*Mel, AAS14450), *Wolbachia *of *Drosophila willistoni *(*w*Will, ZP_01314990), *Ehrlichia canis *(*Eh. c*, AAZ68883), *Anaplasma marginale *(*A.m*, AAV86940), and *Escherichia coli *(*E.coli*, NP_414568). Genbank accession numbers follow the abbreviations of each species indicated above. The conserved catalytic residues are indicated in bold font. The predicted signal peptides are labelled in pale colour and the predicted transmembrane domains are marked by black bars above the alignment. Asterisks (*) denote identical residues, double dots (:) denote conserved substitutions based on amino acid biochemical similarity and single dots (.) denote semi-conserved substitution. The similarity rate is derived from the overall amino acid biochemical similarity of the compared proteins.

Putative *lspA *genes were identified from the available genome databases of *w*Bm and *Wolbachia *endosymbionts of *Drosophila *species. The deduced amino acid sequence of the putative *Wolbachia *(including *w*Bm) LspA proteins contain a predicted signal peptide with three transmembrane domains, which are evolutionarily conserved features in LspA proteins (Figure 1). High sequence identity (80-81%)/similarity (88%) was observed between *B. malayi Wolbachia *LspA and *Drosophila Wolbachia *LspA homologs, all containing the five conserved catalytic residues [21], although a low amino acid identity (25%)/similarity (49%) was revealed when compared to the *Escherichia coli *LspA homolog (Figure 1). The conservation of the amino acids required for function suggests that the *w*Bm LspA gene should be functional.

### *w*Bm LspA recombinant clone is active in a complementation test

To confirm activity of LspA, the recombinant clone was tested using a complementation assay in an *E. coli *strain with a *ts *LspA mutant. The point mutation in the *lspA *gene of *E. coli *Y815 strain confers temperature sensitivity to cell growth. At the non-permissive temperature (42°C), cell growth is hindered by the accumulation of prolipoproteins due to inactivation of LspA enzyme [22]. The plasmids pET21-*Ec*LspA.His (PC, positive control), pET21a-*w*BmLspA.His (T1: test 1), pGEX5.1-GST.*w*BmLspA.His (T2, test 2), pET21a (NC1, negative control 1) and pGEX5.1 (NC2, negative control 2) were transformed into *E. coli *strain Y815 for functional complementation tests of growth. NC1, NC2 and T2 transformants did not grow at 42°C, while both PC and T1 grew and formed visual colonies at the non-permissive temperature, confirming the *w*Bm *lspA *gene (T1) is a functional LspA enzyme (Table 1).

**Table 1:** Complementation assays in *E. coli ts LspA *mutant Y815

*E. coli *Y815 *LspA ts *transformants	30°C	42°C
w/pET21a only (NC1)	+	-

w/pET21a-EcLspA.His (PC)	+	+

w/pET21a-wBmLspA.His (T1)	+	+

w/pGEX5.1 only (NC2)	+	-

w/pGEX5.1-GST.wBmLspA.His (T2)	+	-

Actively growing transformants were plated onto LB agar (100 μg/ml ampicillin, 10 μg/ml tetracycline and 0.6 mM IPTG) and incubated at both the permissive (30°C) and the nonpermissive (42°C) temperature for 3 days for colony formation as described [21]. '+' means growth, '-' means no growth

### Globomycin resistance assays confirm *w*BmLspA activity

**Figure 2:**
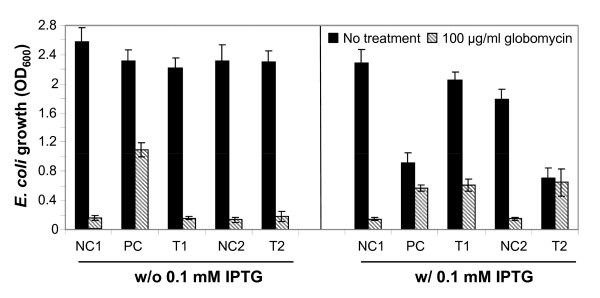
**Globomycin resistance assays of *w*Bm*lspA*-transformed T7 express *E. coli *cells**. Cell growth was measured at 600 nm (OD_600_). NC1: pET21a vector only; PC: pET21a-*Ec*LspA.His; T1: pET21a-*w*BmLspA.His; NC2: pGEX5.1 vector only; T2: pGEX5.1-GST.*w*BmLspA.His. Starting OD is 0.1. Cells grow at 16°C for 16 hours.

**Figure 3:**
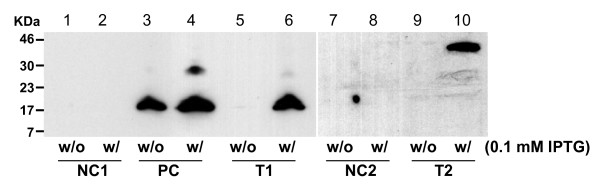
**Expression of recombinant LspA proteins in T7 express *E. coli *with and without IPTG induction**. *w*Bm*LspA *and *EcLspA *transformed *E. coli *cell lysates (~ 200 μg total protein/well) were loaded. The expressed recombinant LspA proteins were detected with Mouse anti-His monoclonal antibody (details in methods and materials). For each construct of samples without (w/o) and with (w/) IPTG induction are shown, Lane 1 & 2: *E. coli *cell/pET21a (NC1), Lane 3 & 4: *E. coli *cell/pET21a-*Ec*LspA.His (PC), Lane 5 & 6: *E. coli *cell/pET21a-*w*BmLspA.His (T1), Lane 7 & 8: *E. coli *cell/pGEX5.1 (NC2), Lane 9 & 10: *E. coli *cell/pGEX5.1-GST.*w*BmLspA.His (T2). The predicted molecular weights of recombinant *w*BmLspA.His, *Ec*LspA.His and GST.*w*BmLspA.His proteins are 19, 20 and 44 kDa, respectively.

Globomycin resistance assays in *E. coli *were conducted to further test the function of the *w*Bm *lspA *gene. LspA inhibition by globomycin leads to the accumulation of unprocessed prolipoproteins in the inner cell membrane and thus hinders cell growth [16,23,24], while the inhibition can be overcome by over-expression of a functional *lspA *gene from other bacterial sources, which confers globomycin resistance [21,25-27]. To confirm this with the *w*Bm *lspA *gene constructs, the plasmids NC1, PC, T1, NC2 and T2 in T7 Express *E. coli *were tested in globomycin resistance assays. With one exception, in the group without IPTG induction, all transformants grew well in the absence of globomycin and showed arrested growth when treated with 100 μg/ml globomycin. The exception was the PC transformant (*E. coli *transformed with pET21a-*Ec*LspA.His), which conferred globomycin resistance in the absence of induction by IPTG (Figure 2). The significant "leaky" expression of *Ec*LspA.His was also detected by western blot analysis (Figure 3). In the IPTG induction set, the cell growth in both PC and T2 transformants (*E. coli *transformed with pGEX5.1-GST.*w*BmLspA.His) was dramatically inhibited even without addition of globomycin, suggesting that a relatively high level of overexpression of *Ec*LspA.His and GST.*w*BmLspA.His proteins alone could hinder normal cell growth. *w*BmLspA.His (T1) was expressed when induced with IPTG, but did not lead to the significant growth inhibition observed for PC and T2 transformants. In the IPTG induction group, when treated with 100 μg/ml globomycin, *E. coli *cells with overexpressed *Ec*LspA.His (PC), *w*BmLspA.His (T1) or GST.*w*BmLspA.His (T2) conferred strong globomycin resistance compared to negative controls (NC1 and NC2) (Figure 3). A synthetic *w*Bm *lspA *gene was created for improving gene expression level in both *E. coli *Y815 and T7 Express *E. coli*. However, no detectable improvement was observed in both *E. coli *cell strains (data not shown).

### Globomycin affects *Wolbachia *growth in a cell-based assay

**Figure 4:**
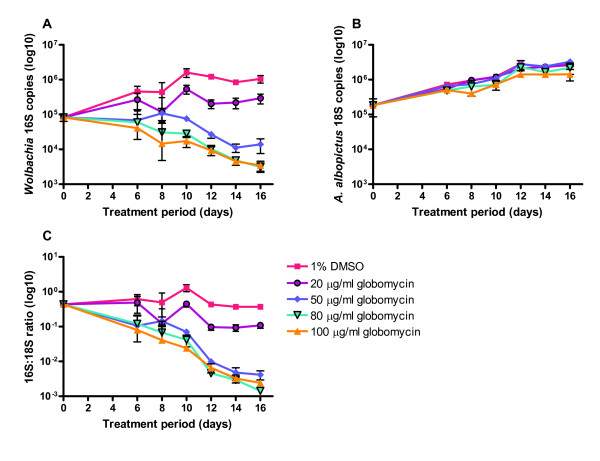
**Dynamics of *Wolbachia *and cell response to globomycin over 16 days**. *Wolbachia *growth was measured by qPCR targeting the 16S rRNA gene (A), C6/36Wp cell growth was measured by targeting the 18S rRNA gene (B) and data was normalized by calculating the ratio of 16S copies to 18S copies (C). Error bars represent standard deviations from triplicate cultures.

We investigated whether globomycin affected *Wolbachia *growth in a cell-based assay using *Wolbachia*-infected insect cells (C6/36Wp; [28]). Cultures were treated with globomycin in a 96-well plate format and *Wolbachia *growth was determined by qPCR targeting the *Wolbachia *16S rRNA gene. As shown in Figure 4A, *Wolbachia *16S copy numbers were significantly reduced when compared to the DMSO (1%) control (*P *< 0.01). This reduction was dose-dependent and equated to log reductions of 1.33, 1.75 and 1.97 for 50, 80 and 100 μg/ml concentrations respectively at day 10 after initiation of treatment, which increased to between 1.88 and 2.48 by day 16. The reduction in 16S gene copy numbers achieved with globomycin at 20 μg/ml, although significant (*P *< 0.05), did not extend beyond a 0.55 log-drop throughout the test period. *A. albopictus *18S rRNA gene copy numbers were also analyzed by qPCR in order to check for any effect of globomycin on cell growth (Figure 4B) and to normalize the 16S data by producing ratios of 16S to 18S gene copy numbers (Figure 4C). Globomycin had no significant effect on cell growth at concentrations up to and including 80 μg/ml (Figure 4B), although 100 μg/ml globomycin did have a small but significant effect on cell growth (*P *< 0.05). The normalization of 16S copy numbers to 18S copy numbers did not affect the overall results (Figure 4C).

### Globomycin affects lipoprotein processing

**Figure 5:**
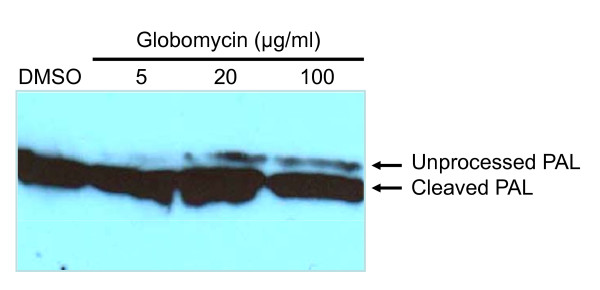
**Inhibitory effect of globomycin on *Wolbachia *PAL processing**. C6/36Wp cells were incubated with three concentrations of globomycin or DMSO control for 24 hours and extracts were subjected to Western blotting using antibodies to *Wolbachia *PAL. Cells treated with globomycin have 2 PAL proteins detectable, indicating inhibition of full lipoprotein processing.

As globomycin targets the enzyme signal peptidase II in the lipoprotein biosynthesis pathway [16], any inhibition that occurs should prevent the cleavage of the signal peptide and thus result in an accumulation of the prolipoprotein. Cells treated with globomycin were used to assess the effect of this drug on lipoprotein processing in *Wolbachia*. A western blot using antibody raised against recombinant *w*Bm PAL was conducted on lysates of cells treated with globomycin or vehicle-control for 24 hours. As shown in Figure 5, globomycin treatment inhibited processing of the precursor of the *Wolbachia *lipoprotein PAL in a dose-dependent manner. When compared to the vehicle control which shows a band of approximately 15 kDa representing the mature lipoprotein, the globomycin treated cells also showed an additional larger band of approximately 18 kDa. This suggests that the target of globomycin in *Wolbachia *is LspA and indicates that the resulting inhibition of growth and death of bacteria is due to the accumulation of prolipoprotein in the cytoplasmic membrane [16,23,24].

### The effect of globomycin on the motility and viability of *B. malayi in vitro*

**Figure 6:**
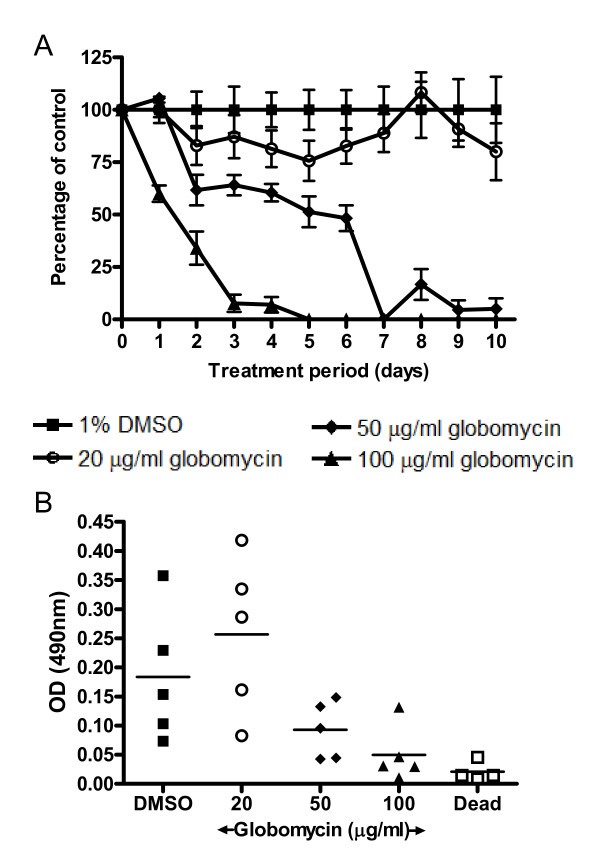
**The effect of globomycin on *B. malayi *motility and viability**. The motility of worms cultured in the presence of globomycin was scored daily and is presented as percentages of the control (1% DMSO) motility (A). At the end of the test period worms were used in an MTT assay to assess effects on nematode viability (B). Remaining worms were used for qPCR analysis of *Wolbachia *numbers per worm (data not shown).

The effect of this compound on nematode motility was examined by incubating *B. malayi *adult females with globomycin *in vitro*. Motility was assessed daily using a method described by Rao and Weil [29]. Over the test period of 10 days, globomycin reduced motility in a dose-dependent manner (Figure 6A). The highest concentration of globomycin (100 μg/ml) rendered the majority of worms inactive by day 3. Globomycin used at a concentration of 50 μg/ml was also found to render the majority of the nematodes immotile by day 7 (Figure 6A). A similar dose dependent reduction in motility was observed in male worms (data not shown).

At the end of the motility test period, worms were collected for use in an MTT assay to assess viability. Figure 6B shows reduced viability of those exposed to the higher concentrations of globomycin, which at a concentration of 100 μg/ml globomycin was significantly reduced compared to vehicle-treated controls at 10 days post-treatment (*P *< 0.05). Furthermore, the MTT result for this treated group was not significantly different from that obtained from dead worm controls (*P *= 0.29) suggesting that globomycin at this concentration is able to kill adult *B. malayi in vitro*.

## Discussion

One of the objectives of the A-WOL consortium is to identify and validate novel drug targets of *Wolbachia *to develop high throughput target based drug screening assays. Validating a potential drug target in *Wolbachia*, however, is problematic as these bacteria are not amenable to genetic manipulation. We have therefore used complementation assays of mutant *E. coli *to validate enzymatic function and a pharmacological inhibitor of lipoprotein biosynthesis, globomycin, to determine the effect of inhibition of lipoprotein biosynthesis on *Wolbachia *in a whole organism cell-based drug screening assay.

*w*Bm contains two genes encoding enzymes of the lipoprotein biosynthesis pathway [15,30]: *lgt*, prolipoprotein diacylglyceryl transferase and *lspA*, lipoprotein signal peptidase. The third enzyme in the pathway responsible for triacylation, typically found in other bacteria, appears to be absent from *Wolbachia*. Although *Wolbachia *is intractable to genetic manipulation we were able to confirm that *Wolbachia *LspA is functional in *E. coli*, despite having only 25% identity and 49% amino acid similarity to its *E. coli *homolog. *w*Bm LspA has retained the conserved catalytic residues required for function and can genetically complement deficient growth of an *lspA ts E. coli *mutant. Globomycin is a known inhibitor of LspA and overexpression of *Wolbachia *LspA in wild-type *E. coli *conferred resistance to this compound allowing us to conclude that the expressed gene was functional.

We used a *ts E. coli LspA *mutant to confirm the functionality of the *w*BmLspA gene (Table 1). As expected, the N-terminal GST-fused full-length *w*BmLspA.His (T2) cannot rescue the *ts *mutant (Table 1); likely due to the loss of signal peptide at its N-terminus, indicating that proper subcellular localization of LspA is crucial for its functional activity. However, the misplaced GST.*w*BmLspA.His still conferred strong globomycin resistance, although its overexpression appeared to be toxic to the *E. coli *cell even without addition of globomycin (Figure 2). This may imply that the mis-localized GST.*w*BmLspA.His (T2) still maintains a structural specificity for globomycin binding. However, after removal of the signal peptide, GST-fused mature *w*BmLspA.His totally lost resistance to globomycin (data not shown).

The leaky expression of *Ec*LspA.His (PC) conferred strong resistance to globomycin without significantly reducing *E. coli *cell growth, while its excessive overexpression, induced by IPTG, although still conferring globomycin resistance, led to striking cell growth inhibition (Figure 2). This might suggest that the expression of LspA is required and at the same time strictly regulated for achieving normal cell growth, which is in line with the fact that codon-optimized *w*Bm *LspA *gene did not improve its expression in *E. coli*.

Lipoproteins perform multiple essential structural and functional roles in bacteria. For example, the lipoprotein PAL is not only involved in the maintenance of outer membrane integrity [31] but has also been demonstrated to be involved in the uptake of nutrients across the membrane of *Pseudomonas putida *[32] and required for virulence in *Haemophilus ducreyi *infection of humans [33]. Therefore the inhibition of lipoprotein biosynthesis is likely to affect multiple functions in bacteria. Globomycin is a 19-membered depsipeptide antibiotic isolated from actinomycetes strains [18-20], which targets LspA. Although not commercially available for use as an antibiotic, it has been widely used as a research tool to determine the requirement of lipoprotein biosynthesis for a range of biological features of bacteria [34,35]. In addition to using globomycin to assess the functionality of the *Wolbachia *LspA enzyme in transformed *E. coli*, we used this compound to assess the importance of the lipoprotein biosynthesis pathway in a *Wolbachia *cell line (C6/36Wp) and in *B. malayi *adult nematodes cultured *in vitro*.

The C6/36Wp system is currently the primary screen used in the A-WOL drug discovery program for *Wolbachia *http://www.a-wol.com, and aims to discover novel compounds with anti-*Wolbachia *activity. In this study, globomycin was shown to have a dose-dependent anti-*Wolbachia *effect on the C6/36Wp cell line, demonstrating a new class of antibiotic active against *Wolbachia*.

Globomycin acts by an inhibition of a prolipoprotein processing enzyme, resulting in an accumulation of the prolipoprotein in the cell envelope [16,23,24] and this inhibition occurs through the non-competitive binding of globomycin to the lipoprotein signal peptidase and not by binding to the substrate [16]. Treatment of *Wolbachia *cell cultures with globomycin resulted in the accumulation of prolipoprotein suggesting a similar mode of action occurs in *Wolbachia*. Furthermore, globomycin affected both the processing of OmpA in *Ehrlichia chaffeensis*, an obligate intracellular bacterium closely related to *Wolbachia pipientis*, and its ability to infect HL-60 cells [35].

Globomycin was also shown to have adverse effects on the motility and viability of adult female and male *B. malayi *nematodes *in vitro*. QPCR analysis did not, however, demonstrate any differences in *Wolbachia *load between treated groups (data not shown) suggesting that the *Wolbachia *qPCR assay may not have sufficient sensitivity to detect effects on *Wolbachia *load over this time frame in nematodes, perhaps due to the slower growth rate of nematode *Wolbachia *compared to insect *Wolbachia *in cell culture. Notably doxycycline also fails to produce a reduction in *Wolbachia *load over this time frame. An alternative explanation is that inhibition of *Wolbachia *lipoprotein biosynthesis is sufficient to affect nematode motility and viability independent of or prior to any effect on *Wolbachia *load. We also cannot rule out a direct effect of globomycin on nematode motility and viability and alternative mechanisms of action have been suggested in *Mycobacterium tuberculosis *[36], where globomycin was found to be active against this bacterium independently of its effect on LspA.

## Conclusions

In summary, in the search for new anti-wolbachial drugs, we have identified and validated the lipoprotein biosynthesis pathway as a target for *Wolbachia *drug discovery and development and shown that globomycin, a drug targeting lipoprotein signal peptidase, is active against *Wolbachia*.

## Methods

### Globomycin

Globomycin was obtained as a kind gift from Professor Masatoshi Inukai of International University of Health and Welfare, Tochigi, Japan. A stock solution of 10 mg/ml was made in dimethylsulphoxide (DMSO) and stored in single-use aliquots at -80°C. Before use, the stock solution was diluted in the appropriate culture medium to the appropriate concentrations.

### *LspA *gene cloning

*B. malayi *DNA (including *Wolbachia *DNA) was extracted with DNeasy extraction kit (Qiagen) from live adult worms purchased from TRS Labs, Athens, GA. Primers were designed with restriction endonuclease sites (Additional File 1; Table S1), based on the available *w*Bm and *E. coli lspA *gene sequences (Genbank accession numbers AE017321 and NC_000913, respectively). The full-length *lspA *genes from *B. malayi Wolbachia *(*w*Bm) and *E. coli *were amplified by PCR using Phusion polymerase (New England Biolabs, NEB), and were cloned into the expression vector pET21a+ with a C-terminal 6XHis tag (Novagen) after digestion with corresponding restriction endonucleases (NEB). The generated plasmids were named as pET21a-*w*BmLspA.His and pET21a-*Ec*LspA.His, respectively. *w*Bm*lspA *gene with an added C-terminal 6XHis tag was also cloned into the expression vector pGEX5.1 (GE Healthcare) with a N-terminal GST fusion, named as pGEX5.1-GST.*w*BmLspA.His. A codon-optimized version of *w*Bm*lspA *was designed by DNAworks oligonucleotide designing software [37], synthesized using USER cloning methods [38] and cloned into pET21a+ vector for improvement of gene expression. The T7 Express competent *E. coli *strain 2566 (NEB) was used as a host for plasmid recipients. The sequences of the *lspA *gene inserts were verified by DNA sequencing.

### Recombinant LspA protein expression in *E. coli*

Expression of C-terminally 6XHis tagged *w*BmLspA, *Ec*LspA, and GST.*w*BmLspA recombinant proteins in T7 Express *E. coli *strain with and without isopropyl-beta-D-thiogalactopyranoside (IPTG) inductions was detected by Mouse anti-His tag monoclonal antibody and subsequent secondary horseradish peroxidase (HRP) conjugated Rabbit anti-Mouse IgG antibody (Novagen) in combination with the use of LumiGLO^(r) ^chemiluminescent reagent and peroxide (Cell Signaling Technology, CST).

### *ts E. coli lspA *mutant

*E. coli *strain Y815 is a type II lipoprotein signal peptidase [21] temperature sensitive (*ts*) mutant, containing a pHY001 vector, which carries a tetracycline resistance gene (*tet^R^*) and a *lpp *gene encoding a major outer membrane lipoprotein inducible by IPTG [22].

### Complementation assays in *E. coli*

The constructs pET21a-*w*BmLspA.His, pET21a-*Ec*LspA.His, and pGEX5.1-GST.*w*BmLspA.His, along with the vector only controls, pET21a and pGEX5.1, were transformed into *E. coli *Y815 cells with appropriate antibiotic selection. The *E. coli *Y815 *ts lspA *mutant grows normally at 30°C, but not at 42°C for colony formation. The detailed protocol is described in [21].

### Sequence analysis of *Wolbachia lspA *genes

The LspA protein sequences from different *Wolbachia *strains and other reference species (shown in Figure 1) were retrieved from NCBI Genbank database [39] via protein-protein BLAST similarity searches and were aligned using CLUSTALX 1.83 [40]. The sequences in the alignment were further analyzed by Genedoc 2.6 [41] for calculating percentage of amino acid identity/similarity, TMHMM 2.0 [42] for prediction of transmembrane domains, and SignalP 3.0 [43] for predicting signal peptide.

### Globomycin resistance assays in *E. coli*

The experiments were devised and developed from a previous strategy [21]. T7 Express *E. coli *were transformed with plasmids pET21a-*w*BmLspA.His, pET21a-*Ec*LspA.His, and pGEX5.1-GST.*w*BmLspA.His, along with the vector only controls pET21a and pGEX5.1. Transformants were grown to 0.6 ~ 1.0 OD_600 _at 37°C in Luria-Bertani (LB) medium containing 100 μg/ml ampicillin before being diluted to 0.1 OD_600_. The diluted samples were equally divided into IPTG (0.1 mM) induced and non-induced groups. In each group, the samples were further divided into globomycin treatment (100 μg/ml) and non-treatment subgroups. All samples were grown at 16°C for 16 hours with shaking. The samples with high cell density were diluted before measurement by spectrophotometer at 600 nm for accuracy. The samples had two replicates in each experiment and three independent experiments were carried out in total.

### *Wolbachia *cell-based drug screening assay

An *Aedes albopictus *cell line C6/36 (ATCC number CRL-1660) stably infected with *Wolbachia pipientis w*AlbB (C6/36Wp) was routinely cultured in Leibovitz-15 medium containing 2 mM L-glutamine, 5% foetal bovine serum, 1% non-essential amino acids and 2% tryptose phosphate broth at 26°C [28]. For drug assays, C6/36Wp cells were cultured in 96-well microtitre plates at a concentration of 10,000 cells per well overnight. Globomycin and vehicle (DMSO) controls were added in triplicate at the appropriate concentration, based on previous publications [17,19,21], the following day and replaced every 4 days. Samples were collected at appropriate timepoints by washing once in phosphate buffered saline (PBS) then adding 150 μl Wizard^® ^SV Lysis Buffer (Promega) to each well. Lysates were stored at -80°C for processing at a later date.

### Quantitative PCR (qPCR)

Genomic DNA was prepared from cell lysates using the Wizard^(r) ^SV 96 Genomic DNA Purification System (Promega) according to the manufacturer's instructions and eluted in 100 μl water. qPCR targeting the 16S rRNA gene of *Wolbachia *and the 18S rRNA gene of the cell was conducted according to that described by [44] with minor modifications. Briefly, reaction mixtures consisted of a pair of primers (5'-TTGCTATTAGATGAGCCTATATTAG-3' and 5'-GTGTGGCTGATCATCCTCT-3' for 16S ribosomal DNA qPCR and 5'-CCGTGATGCCCTTAGATGTT-3' and 5'-ATGCGCATTTAAGCGATTTC-3' for 18S ribosomal qPCR) at 200 nM each, 1 × SYBR Green reaction mix (Qiagen) and 2 μl DNA in the case of 16S qPCR or 1 μl DNA for 18S qPCR. Quantitative DNA standards were prepared as serial dilutions from stock single-stranded oligonucleotides representing the full-length amplicons (Sigma Genosys) and used at 5 × 10^6 ^to 5 × 10^0 ^copies [44], in duplicate reactions. Assays were performed on a DNA Engine PTC-200 thermocycler (MJ Research, GRI) with Chromo4 real-time PCR detection system (Bio-Rad) using the following conditions: denaturation at 95°C for 15 min followed by 40 cycles of 95°C for 15 sec, 55°C for 30 sec and 72°C for 15 sec. Melting curve analysis was performed between 50°C and 95°C to confirm specificity. Copy number was calculated from the standard curve by standard methods.

### Western blot analysis

C6/36Wp cells were cultured in duplicate as described for the cell-based drug assays. Following 24 hours of globomycin treatment, cells were washed once in PBS then lysed by adding 25 μl of ice-cold RIPA buffer (Pierce) containing freshly added protease inhibitor solution (GE Healthcare) and incubating at room temperature for 5 min. Duplicate cultures were pooled and centrifuged at 16,000 × *g *for 15 min and the lysates were collected. Cell lysates were diluted in 2 × Laemmli sample buffer containing reducing agents (Sigma Aldrich) and incubated at 96°C for 10 min. 25 μl of samples were subjected to SDS-PAGE using 15% Tris-HCl gels (Bio-Rad) and protein bands were transferred onto a 0.45 μm PVDF membrane. After blocking for 2 h in 4% milk diluted in Tris-buffered saline (TBS) containing 0.1% Tween, membranes were incubated overnight at 4°C in affinity-purified anti-*B. malayi Wolbachia *PAL (*w*BmPAL) antibody [15] diluted 1 in 5,000 in blocking buffer. Membranes were washed at least four times in TBS 0.1% Tween then incubated with secondary antibody (goat-anti-rabbit-HRP, Perkin Elmer) diluted 1 in 5,000 for one hour at room temperature. Following washing, the blots were developed using the Supersignal West system (Pierce).

### *In vitro B. malayi *assays

Adult *B. malayi *were obtained from TRS Laboratories, Athens, Georgia. Female and male adult worms were cultured in 12-well plates, five worms per well, three wells per group in 2.5 ml RPMI containing 10% foetal bovine serum and penicillin-streptomycin (Invitrogen, 200 U/ml/200 μg/ml final concentration). Globomycin and vehicle controls were added the following day and motility was scored daily in a blinded manner using the scoring system described by [29]. The assay was terminated at day 10 and worms were used in an MTT assay to assess viability [45]. Worms were added singly to wells of a 96 well plate and washed with 200 μl PBS. 200 μl MTT was added at a final concentration of 0.5 mg/ml in PBS and the plate was incubated for two hours at 37°C with 5% CO_2_. MTT solution was removed and worms were washed twice with PBS then incubated in 200 μl of DMSO for one hour at 37°C, 5% CO_2 _in order to solubilise the formazan product. The plate was read at 490 nm using DMSO alone as a blank. Frozen *B. malayi *were used as non-viable controls. Remaining worms were stored at -80°C for DNA extraction at a later date.

### Statistical analysis

Differences between groups were assessed using Student's T test.

## Competing interests

The authors declare that they have no competing interests.

## Authors' contributions

KLJ participated in the design of the study, conducted the cell-based assays and qPCR analysis, western blot analysis and drafted the manuscript. BW participated in the design of the study, conducted the cloning and *E. coli *experiments and helped to draft the manuscript. AG performed the *B. malayi *assays and assisted with data analysis. LF participated in the design of the study and assisted with data analysis and interpretation. BES co-designed the study and helped to draft the manuscript. MJT designed and co-ordinated the study and helped to draft the manuscript. All authors read and approved the final manuscript.

## Supplementary Material

Additional file 1 **Table S1 - *Wolbachia *(*w*Bm) and *E. coli LspA *gene specific primers used**. *Wolbachia *(*w*Bm) and *E. coli LspA *gene specific primers were used for PCR amplification of the full-length coding sequences for cloning into pET21a+ vector and pGEX5.1 vector. Primers were designed based on sequence information available in Genbank http://www.ncbi.nlm.nih.gov. Restriction enzyme sites in primers are underlined. Abbreviations used: *w*Bm: *Wolbachia *endosymbiont of *Brugia malayi*, Ec: *E. coli*, f: forward primer, r: reverse primer, LspA: type II lipoprotein signal peptidase. Click here for file
