# Supplementary material for: Lipoprotein biosynthesis as a target for anti-Wolbachia treatment of filarial nematodes
Source: Parasit Vectors. 2010 Oct 14;3:99. doi: 10.1186/1756-3305-3-99 (PMC2964653; doi:10.1186/1756-3305-3-99)
Supplement: Additional file 1 — Table S1 - Wolbachia (wBm) and E. coli LspA gene specific primers used. Wolbachia (wBm) and E. coli LspA gene specific primers were used for PCR amplification of the full-length coding sequences for cloning into pET21a+ vector and pGEX5.1 vector. Primers were designed based on sequence information available in Genbank http://www.ncbi.nlm.nih.gov. Restriction enzyme sites in primers are underlined. Abbreviations used: wBm: Wolbachia endosymbiont of Brugia malayi, Ec: E. coli, f: forward primer, r: reverse primer, LspA: type II lipoprotein signal peptidase. [file 1756-3305-3-99-S1.PPT]

## Slide 1
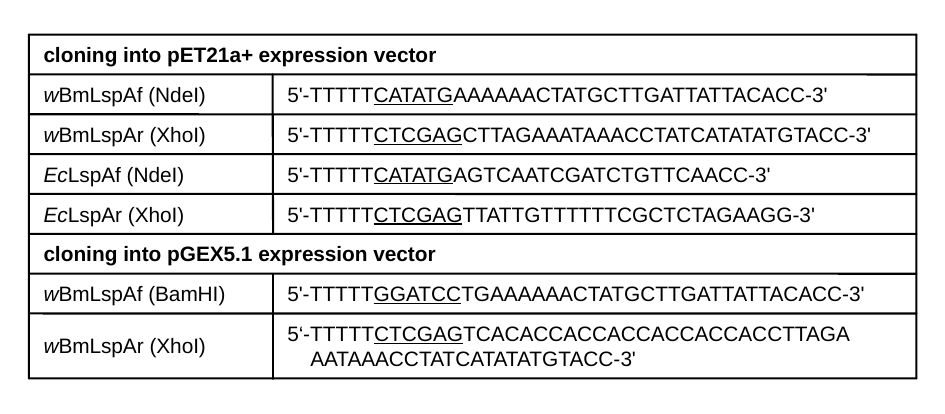

cloning into pET21a+ expression vector
wBmLspAf (NdeI)
5'-TTTTTCATATGAAAAAACTATGCTTGATTATTACACC-3'
wBmLspAr (XhoI)
5'-TTTTTCTCGAGCTTAGAAATAAACCTATCATATATGTACC-3'
EcLspAf (NdeI)
5'-TTTTTCATATGAGTCAATCGATCTGTTCAACC-3'
EcLspAr (XhoI)
5'-TTTTTCTCGAGTTATTGTTTTTTCGCTCTAGAAGG-3'
cloning into pGEX5.1 expression vector
wBmLspAf (BamHI)
5'-TTTTTGGATCCTGAAAAAACTATGCTTGATTATTACACC-3'
5‘-TTTTTCTCGAGTCACACCACCACCACCACCACCTTAGA
 AATAAACCTATCATATATGTACC-3'
wBmLspAr (XhoI)
